# The impact of social support, physical and psychological performance on sleep outcomes in Iranian older adults: a case-control study

**DOI:** 10.1186/s12877-023-04455-3

**Published:** 2023-12-01

**Authors:** Zahra Salehi, Hajar Pasha, Seyed Reza Hosseini, Farzan Kheirkhah, Ali Bijani

**Affiliations:** 1 Student Research Committee, Babol University of Medical Sciences, Babol, Iran; 2 Social Determinants of Health Research Center, Health Research Institute, Babol University of Medical Sciences, Babol, Iran; 3 Infertility and Reproductive Health Research Center, Health Research Institute, Babol University of Medical Sciences, Babol, Iran

**Keywords:** Aged, Physical performance, Social support, Sleep, DSSI, PASE, ADL, IADL, MMSE, PSQI

## Abstract

**Background:**

Sleep quality is one of the most important factors to improve the quality of life in older adults and physical and mental health plays an essential role in better sleep quality. This study aimed to determine the impact of social support, and physical and psychological performance on sleep outcomes in Iranian older adults.

**Methods:**

In this case-control study, 400 elder people, who were exposed to sleep problems, and 400 people without sleep problems were randomly selected during 2016–2017 in Amirkola, Iran. Subjects in the case and control groups were matched in terms of gender and age. The demographic characteristics, Duke Social Support Questionnaire (DSSI), Physical Activity Scale for the Elderly (PASE), Activity of Daily Living (ADL), Instrumental Activity of Daily Living (IADL), Mini-Mental State Examination (MMSE), and Pittsburgh Sleep Quality Questionnaire (PSQI) questionnaires were used to collect data. T-test, Chi-square, Pearson Correlation coefficient, and multiple Logistic regression were used for data analysis.

**Results:**

The mean score of DSSI and its domains including social interaction (DSSI.Int) and social satisfaction (DSSI.Sat) were 28.15 ± 3.55, 9.31 ± 1.23, and 18.84 ± 2.88 in the case group and 28.87 ± 3.20, 9.48 ± 1.10, and 19.83 ± 2.44 in the control group, respectively. In this study, the mean scores of MMSE, PASE, ADL, and IADL were 25.36 ± 3.95, 101.71 ± 56.99, 13/97 ± 0.37, 20.59 ± 2/79; respectively. There was a significant inverse correlation between poor sleep quality with DSSI score (rho = -0.165, *P* < 0.0001), DSSI.Int (rho = -0.113, *P* < 0.001), DSSI.Sat (rho = -0.160, *P* < 0.0001), PASE (rho=-0.160, *P* < 0.0001), and IADL (rho = -0.112, *P* < 0.001) score. Therefore, more social support and physical activity improved the quality of sleep. There was a significant negative relationship between DSSI, and its domains with sleep quality in terms of gender. DSSI (rho = 0.25, *P* < 0.0001), DSSI.Int (*P* < 0.0001, rho=-0.18), and DSSI.Sat (*P* < 0.0001, rho=-0.22) was significant in men but not in women. The results of the adjusted logistic regression revealed a significant association between sleep quality problems and DSSI (*p* < 0.045, OR = 1.40), the use of hypnotic drugs (*p* < 0.0001, OR = 7.56), and occupation (*p* <0.03, OR= 12.66).

**Conclusions:**

The results of the present study suggest that low social support and all its domains, PASE, IADL, and using hypnotic drugs may play a role in the development of sleep problems. It can be used as an effective, safe, and low-cost strategy for promoting sleep quality in older adults.

## Introduction

 Aging is one of the inevitable periods of human life and the world’s population is aging rapidly. One of the problems that arise in parallel with old age is sleep disturbance [1]. The definition of sleep disorders is deficiencies in the quality and quantity of sleep, which affect the continuity of sleep [2]. A sleep disturbance is a medical disorder of sleep patterns. Sleep disturbances are classified into dyssomnias, parasomnias, circadian rhythm sleep disorders involving the timing of sleep, and other disturbances including ones caused by psychological or medical conditions [3].

Insomnia is the most common sleep disorder in older adults. It can be defined as difficulty initiating sleep, early morning awakening, daytime sleepiness, difficulty maintaining sleep, and significant functional impairments [4]. The global prevalence of insomnia symptoms is about 30−35%; the prevalence of insomnia disorders ranges from 3.9 to 22.1%, depending on the diagnostic criteria used [5].

Currently, in the Iranian elderly society, difficulty falling asleep and not sleeping well is a health concern. Irregular sleep-wake patterns and inadequate sleep are worrisome in Insomnia is the most common sleep disorder in older adults [6]. The previous study found that 86.1% of older adults suffer from sleep disorders [7]. Some sleep disturbances can be severe enough to interfere with normal social, physical, mental, and emotional functioning. A review of the literature showed that sleep difficulties, deterioration in sleep quality, and sleep disturbances can increase the risk of behavioral problems [2]. Sleep disorders have important negative short- and long-term health consequences. Poor sleep quality may be a predictor of chronic diseases, such as cardiovascular disease, diabetes, obesity, and psychopathology [8]. Also, it adversely affects physical activities and is associated with poor health, reduced daily activities, impaired physical performance, decreased well-being, reduced quality of life, and increased risk of psychological performance such as depression, anxiety, and stress in older adults [9, 10]. In addition, sleep fragmentation is associated with a 22% annual cognitive decline, and poor sleep is also a risk for cognitive impairment in older adults [11].

Although changes in sleep patterns can be considered part of the normal aging process; however, many of these disorders may be associated with pathological etiology that is not a normal part of aging. Numerous factors can lead to sleep disorders, ranging from environmental factors to lifestyle and other medical conditions. Death of spouse/family members, health problems, retirement, changes in circadian rhythm, decreased physical and psychological performance, and poor social support can contribute to sleep disorders in a large percentage of older adults [12].

Studies have shown that social support can be introduced as a predictive factor for all aspects of human health, quality, and life satisfaction. Social support is defined as the level of affection, companionship, and attention of family, friends, and others [13]. The reduction of social relationships, and more importantly, the loss of the supportive and emotional umbrella of the family are among the factors affecting poor sleep quality in older adults [14]. Social factors, including social loneliness, are thought to be associated with an increased risk of insomnia. Meanwhile, increasing the level of social support by creating healthy sleep is important [15].

On the other hand, aging is associated with functional decline in organs [16]. Physical activity is considered a main behavioral factor associated with quality of life and a healthy lifespan [17]. The effect of physical activity and sleep on each other is complex and affects each other in physiological and psychological ways. Physical activity usually helps improve sleep, while sleep disorders can reduce the ability to do physical activity and increase related injuries [18].

One of the important challenges of older adults is the disability to perform daily life activities, and the physical limitation [19]. Due to the importance of daily independent living activities for older adults, unfortunately, they face more problems, including a decrease in independence [20]. A review of the literature showed that one-quarter of people, after reaching retirement age cannot do their activities [21], which may affect sleep quality [19].

In addition to poor physical activity, cognitive impairment is one of the most common problems of older adults, representing a wide range of old age-related impairments; So that about 35% of the elderly exhibit varying levels of cognitive impairment [16]. Cognitive disorders impair attention, memory, judgment, problem-solving skills, performing actions, behavioral execution, and sleep [11]. Better sleep quality can help individuals regain energy and is associated with increased positive thoughts, increased positive emotions, and better emotional regulation [22]. Therefore, timely identification of older adults at risk of cognitive impairment and considering the necessary therapeutic and preventive measures to prevent the progression of sleep disturbances seems necessary.

Since most important aspects in improving the health and quality of life of older adults are maintaining independence in physical and cognitive activities and continuing to live actively, which has a potential effect on improving their health and quality of life, and reducing the costs of health care [23], and consideration the prevalence of sleep problems in older adults and its consequences, it is necessary to recognize social support strategies, physical and cognition performance influence on the sleep quality among elder people. To the best of our knowledge, little is known about the impact of social support, and physical and cognition function on sleep quality in older adults. Thus, this research aimed to determine the impact of social support, and physical and psychological performance on sleep outcomes in Iranian older adults.

## Methods

### Study design and settings

A case-control study was carried out on elderly aged 60 years and above who lived in Amirkola, Iran in 2016–2017. Amirkola is a small town in the north of Iran near the Caspian Sea. This study was done to identify factors that may contribute to a sleep problem by comparing elderly who have that condition (the “cases”) with subjects who do not have the condition but are otherwise similar (the “controls”), and compared based on some assumed causal attribute (social support, physical and psychological performance). The study aimed to identify the factors that influence sleep outcomes. Therefore, this study has a twofold objective. The first is to see if social support is associated with sleep outcomes. The second objective is to see the relationship between physical, and psychological performance and sleep problems.

**Fig. 1:**
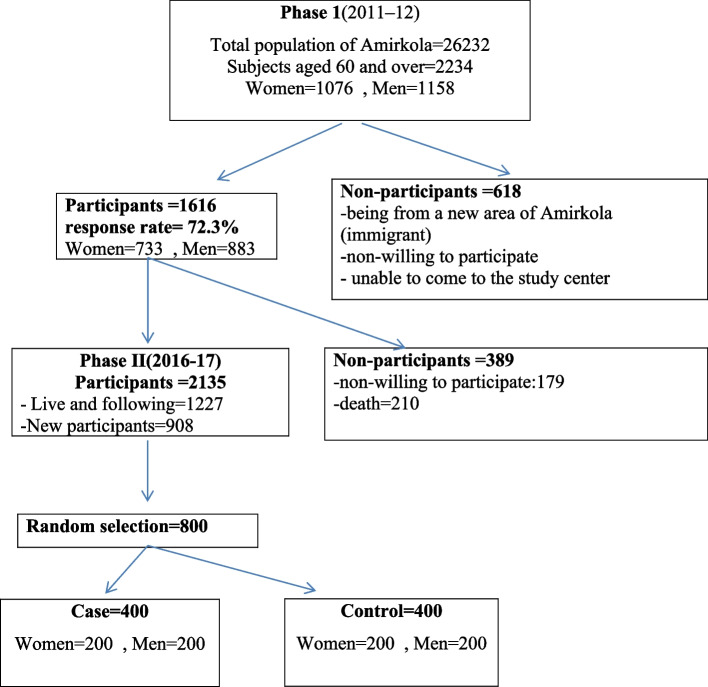
Flow diagram of the data collection process of the AHAP, Iran

The strategy for extracting risk factors included two phases. In the first phase, the original cohort research of AHAP was begun in 2011 on 1616 older adult residents of Amirkola. The total population of Amirkola was 26,232. The total number of elderly people aged 60 years and above was estimated to be 2234. The response rate was 72.3% (1616/2234) in phase I. The second phase of the cohort study was initialed in 2016. All 1616 In the first phase, the original cohort research of AHAP was begun in 2011 on 1616 older adult residents of Amirkola of phase I of the AHAP cohort were followed-up. The number of non-participants was 389 and the new participants were 908. Therefore, 2135 older of aged 60 years and over in Amirkola participated in the study of phase II (Fig. 1). This research is part of the comprehensive protocol to study the health status of older adults in Amir Kola (AHAP = Amirkola Health and Aging Project) in phase 2 AHAP.

The elder people with any of the following criteria were included: informed consent to study, age more than 60 year. and residence in Amirkola. Exclusion criteria included: Unwillingness to participate in the study, Failure to complete the questionnaire, and response to less than 10% of the questionnaire questions.

The main goal of this protocol was to comprehensively assess the sleep outcomes of older adults in the region with an emphasis on social support, physical and psychological performance. Since the exposures were identified after the outcome, we designed a case-control study to demonstrate the effects. In this, the case-control study is concerned with the frequency and amount of exposure (social support, physical and psychological performance) in older adults with sleep problems (cases) and subjects without sleep problems (controls).

### Study population

The participants were evaluated and allocated to one of the following groups:

1. Cases: 400 individuals with sleep problems (using the Pittsburgh Sleep Quality Index (PSQI)).
2. Controls: 400 subjects without sleep problems (The Pittsburgh Sleep Quality cut-off point of ≤ 5 for no sleep problems, and > 5 for those having sleep problems). Controls came from the same population as the cases, but their selection was independent of the exposures of interest.

Therefore, the sample of this study included 800 Iranian people aged 60 and over in Amirkola. A case group was conducted on 400 older adults who were exposed to poor sleep quality; while 400 people were randomly selected in the control group without poor sleep quality. Subjects without sleep problems were matched (ratio 1:1) in terms of gender and age to those with sleep problems.

Since the case-control study is retrospective, there is a possibility of recall bias during the collection of information from older adults. According to the method of collecting information in the form of interviews, it has been tried to minimize this type of bias so that the obtained data have the necessary accuracy. In this study, the subject was randomly selected from older adults in phase II to avoid selection bias. In addition, to avoid Information bias, the investigators were blinded to the aims of the study.

### Selection of cases

All individuals in Amirkola aged 60 years and over in the phase 2 AHAP were invited, and included to participate. Therefore, the general source for selecting cases and controls in this research is the same and based on the population in Amirkola to obtain more accurate results. The cases and controls were selected according to the study entry and exit criteria, and then 400 older adults were randomly selected in the case group with poor sleep quality present study.

### Selection of controls

The controls included individuals aged 60 years and over with a ratio of one to one, who had no sign of sleep problems and were matched in terms of gender and age.

### Sample size and sampling technique

The sample size was calculated, using a power of at least 0.90 and detected an Odds Ratio (OR) of 1.45; the finding showed that 400 older adults were necessary for the case group and 400 for the control group.

### Measurements

After assuring older adults that the information is confidential and obtaining informed consent to enter the study, the demographic characteristics questionnaire and five questionnaires with accepted reliability and validity, including the Pittsburgh Sleep Quality Index (PSQI), Duke Social Support Questionnaire (DSSI), Physical Activity Scale for the Elderly (PASE), Activity of daily living(ADL), Instrumental activity of daily living (IADL), and Mini-Mental State Examination (MMSE) filled out through interviews with older adults in their homes to complete parts of the research questionnaire and then, the next day, the participants came to the Social Determinants of Health (SDH) Research Centre of the University of Babol in Amirkola to complete another questionnaires and examinations. To avoid recall bias and recall bias control method in this study, when older adults in this study do not accurately remember a past event or experience, In addition to the elderly, information was also received from their relatives.

### PSQI

The PSQI consists of 19 self-reported items and 7 subgroups (sleep quality, sleep latency, sleep duration, habitual sleep efficiency, sleep disturbances, taking the hypotonic drug, and daytime dysfunction). On each scale, the individual’s score ranges from 0 to 3. Score 0 = No sleep problem, Score 1 = Moderate sleep problem, Score 2 = Severe sleep problem, and Score 3 = Very severe sleep problem. By obtaining an overall score, this questionnaire can be used to measure overall sleep quality. Range scores vary from 0 to 21, with higher scores indicating poorer sleep quality. A score of ≤ 5 indicates favorable sleep quality, and a total score of > 5 means poor sleep quality. The Cronbach’s alpha = 0.83, and it also had excellent internal consistency [24].

### DSSI

The DSSQ, which was created by Duke in 1984, measures people’s perceptions of the amount and type of social support. The questionnaire has 11 questions and two subscales for social interaction (DSSI.Int) (4 items) and social satisfaction (DSSI.Sat) (7 items), rated using a Likert scale. Social support scores vary between 11 and 33, with higher scores indicating higher levels of social support. The DSSI’s reliability and validity have been confirmed by Goodger B et al., 1999, Internal consistency using Cronbach alpha was 0.77, and Test-retest reliability scores ranged from 0.70 to 0.81 [25]. In this study; the validated Persian version of DSSI was used. Coefficient reliability of Persian questionnaire using Cronbach’s alpha by Faramari et al. (2015) 0.69 calculated the indicator that the internal consistency of this questionnaire is acceptable [26]. To evaluate its validity, the Persian scale was presented to 10 members of the scientific board. The reliability coefficient was obtained as 0.87 and 0.79 respectively after the test-retest (Test-Retest) in 15 seniors [27].

### PASE

Physical activity data were collected using the Standardized Physical Activity Questionnaire for Older Adults (PASE). This questionnaire was first developed by Richard in Boston in 1992 and confirmed its validity and reliability. In Iran, the validity and reliability of this questionnaire were conducted by Ishaghi et al., and Cronbach’s alpha coefficient was found to be 0.97. This questionnaire consists of three parts. The first part is related to free time and contains 6 questions. The second part relates to home activities and has 6 questions; the third part is about work-related activities and has 1 question. There are questions about activities such as walking, bed rest, sedentary sports and leisure activities, and home activities. From this point of view, people were divided into three groups based on their physical activity questionnaire scores: low activity (0–66), moderate activity (67–124), and high activity (> 124). Higher scores indicate more physical activity [28].

### ADL

This scale assessed activities of daily living independently including eating, dressing, walking, bathing or showering, getting in and out of bed or a chair, and using the toilet. Participants answered with the options without help, with a little help, and I was not able to do it. The daily life activities questionnaire was completed to check the ability level in daily life activities for the target samples. This tool has 10 questions and for scoring it, the 0-1-2 method has been proposed. A score of zero shows complete independence (no impairment), while a score of three indicates complete dependence (impairment). It determines the state of independence or dependence in performing daily activities on a scale of 0 (maximum disability and dependency) to 20 (maximum strength and independence), with a higher score indicating less dependence in performing daily activities. A score of 20 means complete independence, a score of 13–19 means partial dependence, a score of 9–12 means partial independence and a score of 0–8 means complete dependence [24]. Taheri et al. (2016) reported Cronbach’s alpha as 0.80 and the intraclass correlation coefficient as 0.76 for ADL [29].

### IADL

This scale evaluated instrumental living activities consisting of the ability to use the telephone, shopping preparing food, do housekeeping, wash personal clothes, travel with vehicles for relatively long distances, take medicine, and manage finances. This tool contains 8 questions. The response spectrum is based on a 5-point Likert. The Likert response is different based on each area. In the area of using the telephone Likert (0–3), shopping Likert (0–3), food preparation Likert (0–3), home activities Likert (0–4), washing clothes Likert (0–2), transportation area of Likert (0–4), drug administration (0–2), and financial management (0–2). The score range is between 0 and 23, where a higher score indicates the greater of a person’s abilities. A score of 23 is interpreted as complete independence, and scores below 23 are interpreted as partial or semi-independence. The validity of the instrument was reported as 0.9. Taheri et al. (2016) revealed Cronbach’s alpha as 0.75 and the intraclass correlation coefficient as 0.79 for IADL [29].

### MMSE

MMSE was used to screen cognitive impairments. This scale assesses the five cognitive statuses of the elderly, namely orientation, information recording, attention and calculation, memory, language, and visual-spatial skills. The score of MMSE ranges between 0 and 30. A score of 25 or higher is considered normal. Scores of 21–24, 10–20, and less than 9 indicate mild, moderate, and severe cognitive impairment, respectively. A lower score indicates worse cognitive function. The reliability coefficient was 0.78 [30].

### Statistical analysis

Data were analyzed using SPSS software version 22 with a *P* value less than 0.05. The qualitative and quantitative data were described as mean frequency (percentage) and mean (standard deviation). The associations of possible risk factors, using multiple logistic regression. In addition, the independent effects of the reproductive variables were evaluated after adjusting for potential confounding variables, including job, income, marital situation, education, and tacking hypotonic drugs using a multiple logistic regression model and the backward selection method. Independent t-test, Chi-square, Pearson correlation coefficient, and multiple logistic regression were used for data analysis. The adjusted OR of the final model was presented using the logistic regression model by a backward approach. Demographic variables were analyzed by descriptive statistics, including percentages for qualitative variables and mean and standard deviation (SD) for quantitative variables.

## Results

The information related to this study was obtained from 800 people aged 60 years and over in Amirkola, Babol. The response rates of the cases and controls were 100%. The majority of the older adults were married (83.75%), homemakers (47.75%), medium income (33.125), illiterate (56.75%), not taking hypnotic drugs (80/13%), no smoking (86.5%), and with a Mean ± SD age of 68.75 ± 6.59. Table 1 summarizes the baseline socio-demographic characteristics of older adults.

**Table 1:** Baseline socio-demographic characteristics of the cases and controls in the AHAP^a^, Iran

Variable	Sleep Quality		***P*** value
	Cases (>5)	Controls (≤5)	
	N (%)	N (%)^b^	
**Gender**			1
Male	200 (50.0)	200 (50.0)	
Female	200 (50.0)	200 (50.0)	
**Age**(years)			1
60-64	120 (30.0)	120 (30.0)	
65-69	120 (30.0)	120 (30.0)	
70-74	80 (20.0)	80 (20.0)	
75-79	60 (15.0)	60 (15.0)	
80-84	10 (2.50)	10 (2.50)	
85-99	10(2.50)	10(2.50)	
**Marital situation**			0.25
Single	71 (17.75)	59(14.75)	
Married	329 (82.25	341(85.25)	
**Educational Level**			0.47
Illiterate	218(54.5)	236(59.0)	
Primary	100(25)	89(22.25)	
Up to diploma and diploma	50(12.5)	51(12.75)	
University	32(8)	24(6)	
**Occupation**			0.17
Unemployed	25(6.25)	26(6.5)	
Homemaker	179(44.75)	203(50.75)	
Worker and farmer	53(13.25)	35(8.75)	
Employed and retired	51(12.75)	41(10.25)	
Self-employed	92(23)	95(23.75)	
**Satisfaction of income**			0.42
very much	1(0.30)	4(1.0)	
Much	6(1.50)	7(1.80)	
Medium	130(32.5)	135(33.8)	
Low	181(45.2)	161(40.3)	
very little	82(20.5)	93(23.3)	
**Tacking hypnotic drugs**			0.0001
No	375(93.75)	266(66.5)	
Yes	25(6.25)	134(33.5)	
**Smoking**			0.67
No	348(50.3)	344(49.7)	
Yes	52(48.1)	56(51.9)	
**Variable**	**Mean(SD)**	**Mean(SD)**	**P value**
**Age**(years)	68.68±6.58	68.81±6.59	0.77

Note: ^a^Amirkola Health and Aging Project. ^b^Values are numbering/percentage (Chi-square and t-test).

As shown in Table 2, there was a significant inverse correlation between poor sleep quality with DSSI score (rho = -0.165, *P* < 0.0001), DSSI.Int (rho = -0.113, *P* < 0.001), DSSI.Sat (rho = -0.160, *P* < 0.0001), PASE (rho= -0.160, *P* < 0.0001), and IADL (rho = -0.112, *P* < 0.001) score. So increasing social support (DSSI, DSSI. Int, DSSI. Sat) and physical activity (PASE, IADL) increased the quality of sleep.

**Table 2:** The correlations between sleep quality with social support and physical activity in the AHAP^a^, Iran

Variables	PSQI	DSSI	DSSI.Int	DSSI.Sat	PASE	ADL	IADL	Age
**PSQI**	1							
**DSSI**	-0.165 0.0001	1						
**DSSI.Int**	-0.113 0.001	0.720 0.0001	1					
**DSSI.Sat**	-0.160 0.0001	0.953 0.0001	0.476 0.0001	1				
**PASE**	-0.160 0/0001	0.248 0.0001	0.262 0.0001	0.200 0.0001	1			
**ADL**	-0.021 0.544	0.200 0.0001	0.219 0.0001	0.157 0.0001	0.120 0.0001	1		
**IADL**	-0.112 0.001	0.527 0.0001	0.585 0.0001	0.412 0.0001	0.433 0.0001	0.287 .0001	1	
**Age**	-0.028 0.429	-0.232 0.0001	-0.215 0.0001	-0.200 0.0001	-0.266 0.0001	-0.059 0.096	-0.421 0.0001	1

Note: ^a^Amirkola Health and Aging Project, Statistical significance was determined by calculating Pearson’s correlational analysis (2-tailed).

There was a significant difference between the two groups in terms of DSSI (*P* < 0.003), DSSI Interaction (*P* < 0.04), DSSI Satisfaction, (*P* < 0.004), PASE (*P* < 0.0001), and IADL (*P* < 0.004). People with poor sleep quality had a lower score in social support and physical activity (Table 3).

**Table 3:** Comparison of social support, physical activity, and mental situation of the cases and controls in the AHAP^a^, Iran

Risk Factors	Sleep Quality			***P*** value
	Case(>5) ***N***=400	Control (≤5) ***N***=400	Total ***N***=800	
	M (SD)^b^	M (SD)	M(SD)	
**DSSI**	28.15±3.55	28.87±3.20	28.51± 3.37	0.003
**DSSI.Int**	9.31±1.23	9.48±1.10	9.39± 1.16	0.04
**DSSI.Sat**	18.84±2.88	19.38±2.44	19.11± 2.66	0.004
**PASE**	93.68±54.51	109.73±59.48	101.71±56.99	0.0001
**ADL**	13.96±0.44	13.97±0.29	13.97±0.36	0.57
**IADL**	20.31±2.95	20.88±2.62	20.59±2.78	0.004
**MMSE**	25.32±3.95	25.39±3.95	25.35± 3.95	0.81

Note: ^a^Amirkola Health and Aging Project. ^a^Values are mean ± SD (T-test)

**Fig. 2:**
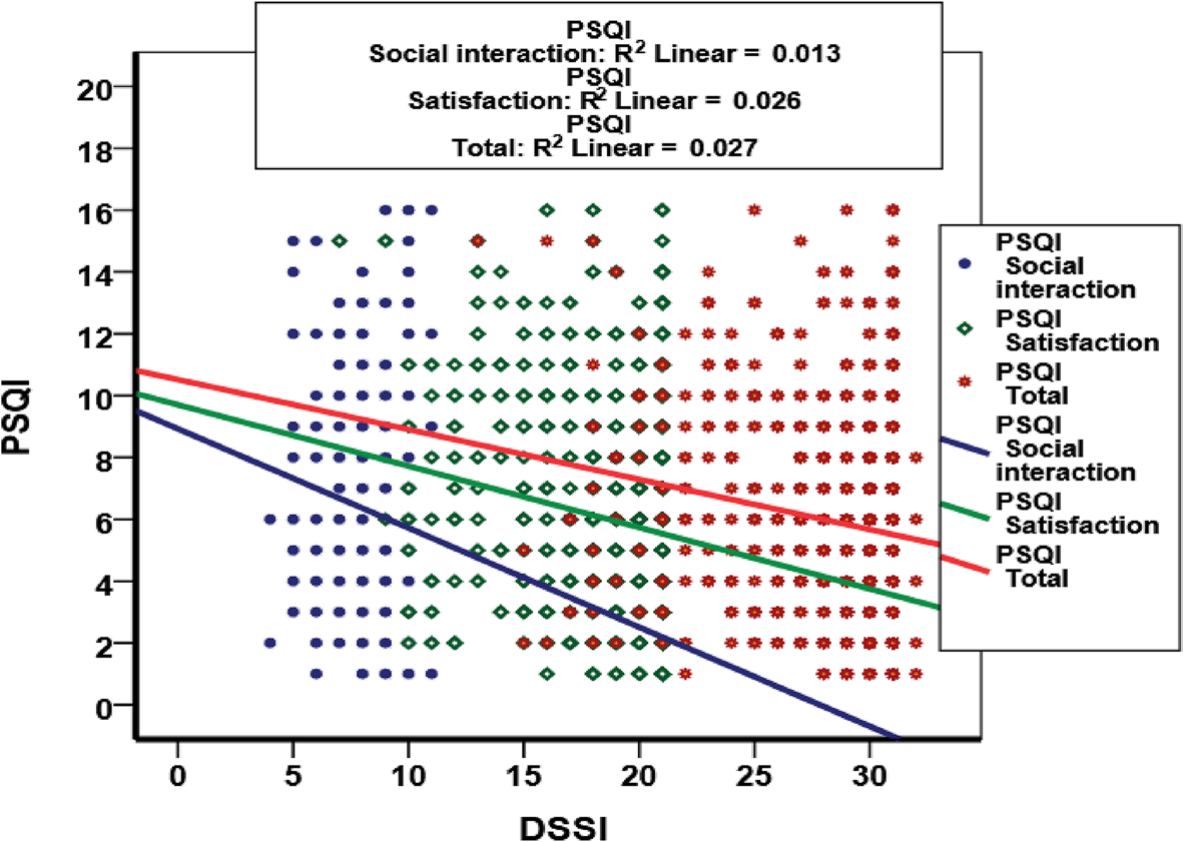
Scatter plot of correlation between sleep quality and social support by its domains among older people in the AHAP^*^, Iran. Note: *Amirkola Health and Aging Project

As shown in Fig. 2, there was a negative significant relationship between DSSI and its domains with sleep quality among older people.

Table 4 presents the outcomes of multiple logistic regression after adjusting for marital Situation, income, education level, job, and taking hypnotic drugs. Taking hypnotic drugs (OR = 7.56, *p* < 0.0001) and less social support (OR 1.40, *p* < 0.045) significantly increased the chances of sleep quality problems (Table 4). The elderly with less social support were 1.4 times more likely to have the risk of poor sleep quality than those with more social support (OR = 1.40, CI = 1.01–1.95).

**Table 4:** Multiple logistic regression analysis to investigate factors affecting the sleep quality of the elder people in the AHAP^a^, Iran, 2016-17

Risk Factors	B	S.E	OR^b^	***P*** value	95%Cl	
					Low	Up
**Marital Situation**						
**Single(R)**			1			
**Married**	0.41	0.23	1.50	0.07	0.96	2.34
**Low Income**	-0.24	0.17	0.78	0.14	0.56	1.08
**Education level**						
Illiterate(R)			1	0.57		
Primary	-0.17	0.21	0.84	0.41	0.55	1.27
Up to Diploma and diploma	0.003	0.28	1	0.99	0.58	1.73
University	-0.42	0.37	0.65	0.25	0.32	1.35
**Job**						
Unemployed(R)			1	0.46		
Housewife	0.01	0.32	1.01	0.96	0.53	1.91
worker and farmer	-0.41	0.39	0.66	0.30	0.30	1.44
Self-employed	-0.15	0.39	0.85	0.69	0.39	1.84
Employee	0.13	0.38	1.14	0.72	0.68	2.39
**Taking hypnotic drugs**						
No(R)			1			
Yes	2.02	0.24	7.56	0.0001	4.76	11.99
**DSSI**						
Yes(R)			1			
No	0.34	0.17	1.40	0.045	1.01	1.95
**ADL**	0.61	.22	1.06	0.78	0.69	1.65
**IADL**	-0.04	0.04	0.96	0.26	0.89	1.03

Note: Sleep quality (Yes; PSQI ≤ 5, No: PSQI >5); Social Support (Yes; DSSI ≥ 30, No: DSSI <30).

^a^Amirkola Health and Aging Project. ^b^OR Odds ratio was calculated using the logistic regression model

Table 5 demonstrates the outcomes of multiple logistic regression in elderly with and without taking hypnotic drugs. The chances of poor sleep quality of the elderly without taking hypnotic drugs were 1.60 times more in persons with less social support than those with more social support (OR = 1.60, CI = 1.16–2.21). Furthermore, The chances of sleep quality problems of the elderly with taking hypnotic drugs were more in employee persons compared to unemployed persons (OR = 12.66, CI = 1.33–119.76).

**Table 5:** Multiple logistic regression analysis to investigate factors affecting the sleep quality of elderly with and without taking hypotonic drugs in the AHAP^a^, Iran, 2016-17

Risk Factors	No taking hypnotic drugs ***N***=641				Taking hypnotic drugs ***N***=159			
	OR	***P*** value	95%Cl		OR^b^	***P*** value	95%Cl	
			Low	Up			Low	Up
**Marital Situation**								
**Single(R)**	1				1			
**Married**	1.57	.05	1.01	2.45	.33	.18	.06	1.69
**Low Income**	.804	.22	.57	1.14	.59	.36	.19	1.83
**Education level**								
Illiterate(R)	1	.36			1	.93		
Primary	.79	.29	.51	1.22	1.37	.62	.39	4.78
Up to Diploma and diploma	1.04	.88	.59	1.86	.84	.85	.15	4.87
University	.59	.18	.27	1.29	1.72	.99	.00	.00
**Job**								
Unemployed(R)	1	.45			1	.23		
Housewife	.86	.65	.44	1.67	2.67	.24	.51	13.92
worker and farmer	.54	.14	.24	1.22	2.22	.46	.26	18.84
Self-employed	.65	.30	.29	1.46	5.50	.13	.60	50.70
Employee	.82	.61	.37	1.78	12.66	.03	1.33	119.76
**DSSI**								
Yes(R)	1				1			
No	1.60	.01	1.16	2.21	1.96	.21	.68	5.64
**ADL**	1.12	.67	.67	1.86	.00	.99	.00	.00
**IADL**	.97	.41	.89	1.05	.92	.44	.74	1.14

Note: Sleep quality (Yes; PSQI ≤ 5, No: PSQI >5); Social Support (Yes; DSSI ≥ 30, No: DSSI <30).

^a^Amirkola Health and Aging Project. ^b^OR Odds ratio was calculated using the logistic regression model

**Fig. 3:**
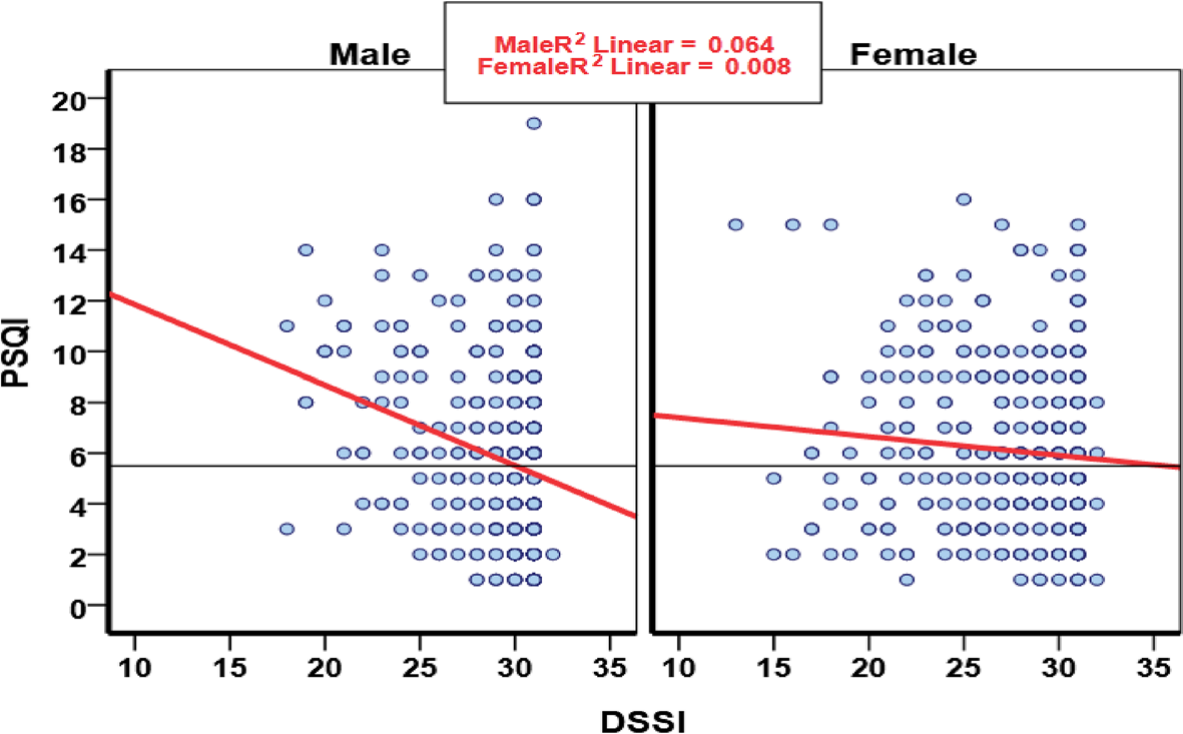
Scatter plot of correlation between sleep quality and social support by gender among elder people in the AHAP^*^, Iran. Note: *Amirkola Health and Aging Project

 There was a negative significant association between DSSI, and its domains with sleep quality in terms of gender. Social support (rho = 0.25, *P* < 0.0001; rho = 0.09, *P* < 0.076), DSSI.Int (rho=-0.18, *P* < 0.0001; rho=-0.06, *P* < 0.27), and DSSI.Sat (rho=-0.22, *P* < 0.0001; rho=-0.09, *P* < 0.067) was significant in men but was not significant in women (Fig. 3).

## Discussion

In the present study, we investigated the association between social support, physical activity, and cognitive performance in terms of their effects on the sleep outcomes of the Amirkola elderly. Although previous research has revealed different risk factors for sleep disorders, as far as we know, it is the first time that the risk factors for prevalent sleep quality have been studied in the Iranian elderly population.

The finding of this study confirmed that social support and its domains are negatively significantly correlated with poor sleep quality. Older adults with low levels of social support had a 1.40 times higher risk of having poor sleep quality compared with individuals with high levels of social support. There was a negative significant relationship between social support, and its domains with poor sleep quality in terms of gender. It was significant in men, but not in women. Furthermore, The chances of poor sleep quality of the elderly without taking hypnotic drugs were 1.60 times more in persons with less social support than those with more social support (OR = 1.60, CI = 1.16–2.21). Consistent with the findings of the current research, Li et al. (2022) found that older men receiving social support have decreased their worry levels and have promoted sleep quality [31]. Another study revealed that individuals who are receiving support from others; can reduce the effects of stress on sleep and improve sleep quality [32]. Greya et al. (2020) found social support to be a major factor influencing sleep quality [33]. Some explanations are suggested for the link between sleep problems and social support deficiency. For example, studies have linked social support deficiency to stress, meaning that the deficiency increases the risk of stress, which in turn, has been revealed to escalate the risk of sleep problems [32]. Another possible mechanism is that social supports are involved in psychological health and thus sleep performance [33]. Also, the difference in the findings in terms of gender may be due to the difference in the circumstances, as women have lower personal income, lower levels of education, more chronic diseases, and more psychological changes (due to hormonal changes caused by menopause. These situations of women can influence this finding.

The current study provides evidence of a high level of the mean score of daily life activities and instrumental activities of daily living among the older adults who did engage in enough physical activity. This finding was in line with the result of a similar study conducted by Habibi Sola et al. (2007) [34]. In this context, Lee et al. (2006) reported that more than 50–60% of older adults reported having no problems performing their daily activities [23]. This finding may put forward the idea that success in aging does not necessarily mean avoiding age-related deficiencies, but rather a continuation to gain more and lose less despite existing obstacles. Although the elderly want to live in an environment free of movement and excitement, it must be acknowledged that by reviving their abilities and proper training in health-promoting behaviors, they can have more physical activity and a better life. This requires careful planning and continuous training [34].

Results of the current study revealed a significant relationship between physical activity (PASE, IADL) with sleep pattern, which was in line with the previous study [35, 36]. A study conducted by Esnaasharieh et al. (2022) demonstrated better sleep quality among participants with more physically active [37]. Another study clarified that physical activity can also prepare the body for a good night’s sleep in older people [28], and the energy stored in the body during sleep can be consumed during physical activity [38]. Studies have also revealed that individuals who engage in more physical activity have had better sleep duration and efficiency, regardless of the intensity or type of activity [36], while Pengpid et al. (2018) highlighted that vigorous PA than moderate PA had no positive effect on sleep quality [39]. One research showed that both very low and very high levels of regular physical activity are correlated with the risk of insomnia [40], and there are controversies about the role of a type of PA. Maybe the difference in the findings is due to the difference in the type and the intensity of physical activity and the age of the subjects. The role of different levels and types of PA on sleep quality still requires being clarified [41]. Possible causes for physical activity’s effectiveness on sleep can involve the autonomic nervous system, endocrine, metabolism, physical functions, and circadian rhythm [42]. Furthermore; poor somatic performance is linked with hypoxia and sleep fragmentation in older adults [43], which can highlight poor sleep [44]. The impact of sleep and physical activity on each other is complex and they interact in physiological and psychological ways. Physical activity usually helps improve sleep, while sleep disorders can reduce the ability to engage in physical activity and increase related injuries [41]. There were some explanations for the relationship between sleep quality and physical activity. Several studies linked physical activity with changes in mood, heart rate, and body temperature. Physical activity increases energy consumption, along with the release of brain-derived neurotrophic factors, melatonin, and serotonin, which in turn have been shown to decrease the risk of insomnia. Therefore, regular physical activity as a non-pharmacological intervention offers numerous psychological and physiological benefits that can be effective in promoting health levels, quality of life, and sleep disorders [42, 45].

The present study depicted normal mean cognitive performance scores in older adults. There was no significant difference in mental state in the two groups, which is consistent with research done by Casavi et al. (2022), where no statistically significant effects were shown for cognitive performance with sleep quality [46]. Mahfouz et al. (2020) also found no statistical difference in sleep quality according to the stress level, and DASS score [7], while Aseem et al. (2021) highlighted a significant link between cognitive performance and sleep quality [47]. The quality of sleep in older adults has been implicated in regulating cognitive function [48]. Sleep has a vital impact on cognitive performance. Lack of sleep can lead to impaired mood and cognitive function. Therefore, the need to improve the health of older adults is increasing [49]. Sleep disturbances are related to various morbidities, typically psychological problems such as depression, and stress, and can lead to dementia [50]. Similarly, Ru et al. (2019) discovered that even a very short nap (15–30 min) effectively improves alertness, task performance, and mood, reducing sleepiness and negative thoughts [51]. Treatment of psychiatric disturbance has a useful effect on sleep quality, and treatment of sleep complaints can lead to improvement in the course of psychiatric disturbance [52]. Maybe the difference in the findings is due to the difference in socio-demographic, lifestyle, and cultural status. Furthermore; enough level of cognitive performance in the elderly adults in this study may lead to improved sleep quality in both groups.

The present study revealed that taking a hypotonic drug was a significant negative predictor of sleep quality, implying that individuals who use hypotonic drugs had poor sleep quality. This finding was in line with the results of the study by Abbaspour et al. (2017) [53], and Lintzeris et al. (2016) [54]. They showed that individuals using soporific drugs compared to normal subjects had poor sleep quality and life quality, While this finding was inconsistent with the study conducted by Satheesh et al. (2020) [44]. Pharmacotherapy is one of the most important therapeutic approaches for sleep disorders. Benzodiazepines are the most commonly used drugs. Although these drugs are highly efficacious, relatively safe, and harmless, they have many side effects that are more common in high-dose, long-term use cases [55]. A review of the literature highlighted high levels of sleep problems in subjects prescribed long-term opioids [54]. More than half of the patients taking sleeping pills had worked up during the night and had trouble falling asleep again. In addition, long-term use of sleeping pills does not affect the return and correction of sleep pattern changes [55]. Sedative-hypnotic drugs are not a good choice and are a safer alternative for older adults with insomnia. With aging, drugs stay in the body longer and are more likely to cause harmful side effects. These medications are also highly addictive. The body becomes used to them over time, which leads to them stopping working. On the other hand, people who take sedative-hypnotic drugs are more likely to have memory problems and drowsy during the day [56]. Therefore; non-medication strategies to address poor sleep can be prioritized [54]. It can also be possible to suggest drug therapy combined with psychotherapy to improve sleep disturbances [53].

Results of the current study revealed the chances of sleep quality problems of the elderly with taking hypnotic drugs were more in employee persons compared to unemployed persons. In contrast to the present study, Najafi et al. (2021) showed that unemployed persons had more chance of poor sleep quality compared to employed patients, which is not consistent with the results of the present study [57]. Ramezanifar et al. (2021) reported that factors affecting sleep problems were job stress, and shift work, which can impair sleep quality. Therefore, therapists are required to attend to occupational factors in intervention and counseling [58].

### Limitations and strengths

The current research had several limitations. Given the fact that seasonal and temporal factors impact the quality of sleep, this study cannot evaluate the quality of sleep over time and prepare objective information concerning sleep, and it only reflects the elderly’ perceptions of sleep and its quality. As that causality of outcome cannot be established in this study design (unless a longitudinal cohort study); therefore it may act as a study limitation, and suggest recommendations for further study to validate the research or further establish associations. Also, the effect of the different cut-off points on scales can have different results. Furthermore, the current study is conducted only in Amirkola Babol, It is suggested that the research be carried out widely not only in Babol City but also in the whole country so that if similar results are obtained, more importance should be given to health-enhancing behaviors in older adults. The strengths of the present research are the different exposures and the high participation rate.

## Conclusion

This research clarified the benefits of physical and psychological approaches on the sleep quality of older adults. The presence of social support and physical activity the older adults had the most significant role in the improved quality of sleep. Therefore, annual screening of older adults regarding health status, and expansion and improvement of the social support network for the elder people may play an effective role in preventing sleep problems.

## Data Availability

The data supported during the present research are available from the corresponding author upon reasonable request.

## Abbreviations

PSQI: Pittsburgh Sleep Quality Questionnaire
DSSI: Duke Social Support Questionnaire
DSSI.Int: Social Interaction
DSSI.Sat: Social Satisfaction
PASE: Physical Activity Scale for the Elderly
ADL: Activity of Daily Living
IADL: Instrumental Activity of Daily Living
MMSE: Mini-Mental State Examination
